# Emergence of californium as the second transitional element in the actinide series

**DOI:** 10.1038/ncomms7827

**Published:** 2015-04-16

**Authors:** Samantha K. Cary, Monica Vasiliu, Ryan E. Baumbach, Jared T. Stritzinger, Thomas D. Green, Kariem Diefenbach, Justin N. Cross, Kenneth L. Knappenberger, Guokui Liu, Mark A. Silver, A. Eugene DePrince, Matthew J. Polinski, Shelley M. Van Cleve, Jane H. House, Naoki Kikugawa, Andrew Gallagher, Alexandra A. Arico, David A. Dixon, Thomas E. Albrecht-Schmitt

**Affiliations:** 1 Department of Chemistry and Biochemistry, Florida State University, Tallahassee, Florida 32306, USA; 2 Department of Chemistry, The University of Alabama, Tuscaloosa, Alabama 35487, USA; 3 National High Magnetic Field Laboratory, Tallahassee, Florida 32310, USA; 4 Chemical Sciences and Engineering Division, Argonne National Laboratory, Argonne, Illinois 60439, USA; 5 Nuclear Materials Processing Group, Oak Ridge National Laboratory, Oak Ridge, Tennessee 37830, USA; 6 National Institute for Materials Science, Tsukuba, Ibaraki 305-0047, Japan

## Abstract

A break in periodicity occurs in the actinide series between plutonium and americium as the result of the localization of 5*f* electrons. The subsequent chemistry of later actinides is thought to closely parallel lanthanides in that bonding is expected to be ionic and complexation should not substantially alter the electronic structure of the metal ions. Here we demonstrate that ligation of californium(III) by a pyridine derivative results in significant deviations in the properties of the resultant complex with respect to that predicted for the free ion. We expand on this by characterizing the americium and curium analogues for comparison, and show that these pronounced effects result from a second transition in periodicity in the actinide series that occurs, in part, because of the stabilization of the divalent oxidation state. The metastability of californium(II) is responsible for many of the unusual properties of californium including the green photoluminescence.

## Abstract (web summary)

The chemistry of the post-plutonium actinides is thought to resemble lanthanides in that bonding is primarily ionic. Here, the authors show that a californium(III) complex displays significantly different properties to those predicted for the free ion owing to a second break in actinide periodicity.

## Graphical abstract

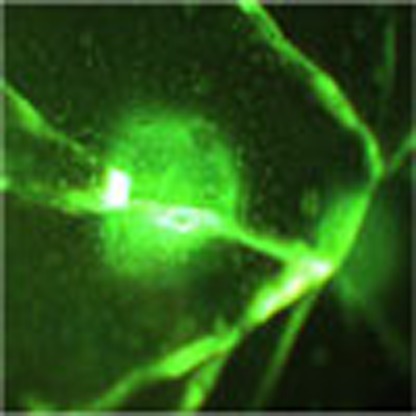

Advances in theory coupled with sophisticated spectroscopic and structural analyses of actinide complexes and materials have transformed the way in which we view these elements from what was once considered mundane to utter fascination^1^,^2^,^3^,^4^. While tantalizing evidence that 5*f* elements might utilize their valence orbitals in bonding was uncovered shortly after the Manhattan Project in the 1950s (ref. 5), it was not until more compelling techniques were applied to this problem that the broader chemical community started to become aware that a simplistic description of the 5*f* series as being ostensibly the same as that of lanthanides is indefensible. Among the more convincing probes of the nature of bonding in these compounds is ligand K-edge XANES^6^, which when coupled with advanced electronic structure modelling has revealed that the metal–ligand interactions can be quite similar to those of *d* orbital interactions in transition metal coordination complexes^2^,^7^. However, the majority of this progress is restricted to early actinides for quite practical reasons that include a lack of structural and spectroscopic data from mid- to late actinides, and increasing difficulties in quantum mechanical calculations that come with the larger number of 5*f* electrons and variety of available acceptor orbitals that include the 6*d*, 7*s* and 7*p*.

Californium is the last element in the periodic table where it is possible to measure the properties of a bulk sample; albeit these measurements come with considerable experimental challenges^8^. Nevertheless, one of the hallmarks of the nuclear era was the maturation of ultramicrochemical techniques that had been perfected by the time californium became available in appreciable quantities. This enabled characterization of binary compounds such as halides and oxides^9^, and even in these systems peculiarities were observed that include abnormally broad *f*-*f* transitions in electronic absorption spectra, and consistently reduced magnetic moments with respect to that calculated for the free ion^10^,^11^. A testament to the unexpected features of californium compounds is that CfCp_3_ (Cp=cyclopentadienyl) has a deep red colouration instead of the expected bright green^12^. These deviations from isoelectronic Dy(III) compounds point to changing chemical behaviour and alterations in electronic characteristics as the result of complexation that are not paralleled by lanthanides or, more importantly, by lighter actinides.

There are several lines of reasoning that shed some light on the departure of electronic behaviour late in the actinide series. The first of these arguments is electrochemical (that is, thermodynamic). The increasing stability of the 3+ oxidation state among heavier 5*f* elements is typically ascribed to the contraction, localization and lowering in energy of the 5*f* orbitals, such that by americium oxidation states beyond 3+ are difficult to achieve. What is seldom recognized is that the divalent oxidation state is also becoming increasingly thermodynamically accessible late in the actinide series^13^. In fact, the solution chemistry of nobelium could not be explained until it was determined that unlike earlier 5*f* elements, its most stable oxidation state in aqueous media is 2+ because ostensibly this provides a closed-shell 5*f*^14^ configuration^14^. Californium is the first element in the actinide series where the 2+ oxidation state is chemically accessible at reasonable potentials, and, in fact, Cf(II) compounds, such as CfCl_2_, have been successfully prepared and characterized^15^.

Further support for the stability of Cf(II) comes from ambiguity in the valency of metallic californium. While the connection of the behaviour of elements in their metallic state with that of their ions in solution may seem tenuous, in this case many parallels can be discerned. Early reports indicated that californium acted as a divalent metal much like europium and ytterbium^16^,^17^. However, it is now understood that thin films of californium trap a metastable divalent state, whereas the bulk material is trivalent as expected^18^. Similar behaviour is found with samarium, which possesses a reduction potential similar to that of californium. The coupling of these two lines of evidence indicates that californium is located at an electronic tipping point in the actinide series, and it plays a role much like that of plutonium where an earlier departure in 5*f* character takes place. In the case of plutonium, its unique electronic properties that stem from the changing roles of the 5*f* orbitals allow it to undergo six phase transitions before melting, and it can simultaneously equilibrate four oxidation states in solution; both of these features are unmatched anywhere else in the periodic table.

We recently reported on the preparation, structure, properties and results of quantum chemical calculations of Cf[B_6_O_8_(OH)_5_] (ref. 8). The structure of this compound is not paralleled by the lighter actinides, and, more importantly, the electronic properties are rather unexpected from an *f* element. These unusual features include broad *f*-*f* transitions, strongly vibronically coupled photoluminescence and a massive reduction in the magnetic moment with respect to that calculated for the free ion. All of these features point to complexation perturbing the 5*f* orbitals in a way that is more typical of ligand-field effects on transition metal ions. In fact, density functional theory (DFT) and multi-reference molecular orbital calculations support the donation of electron density from the borate ligands into the 5*f* orbitals, as well as the 6*d*, 7*s* and 7*p*, and the latter method reveals strong ligand-field splitting that is among the largest observed for an *f* element.

However, this prior study^8^ opened up as many questions as it answered. First, are these perturbations of the ground and excited states of Cf(III) unique to the highly electron-rich environment that borate provides, or are these effects achievable with much simpler and better understood ligands? Second, is it possible that indicators of these effects have been observed since the earliest developments of californium chemistry (*vide supra*), but simply not recognized for their significance? In the present study, we answer these questions and provide a hypothesis to explain why this chemical behaviour is not observed earlier in the actinide series, and why we predict that these effects will only become more pronounced later in the heaviest 5*f* elements.

## Results

### Synthesis and characterization

**Figure 1:**
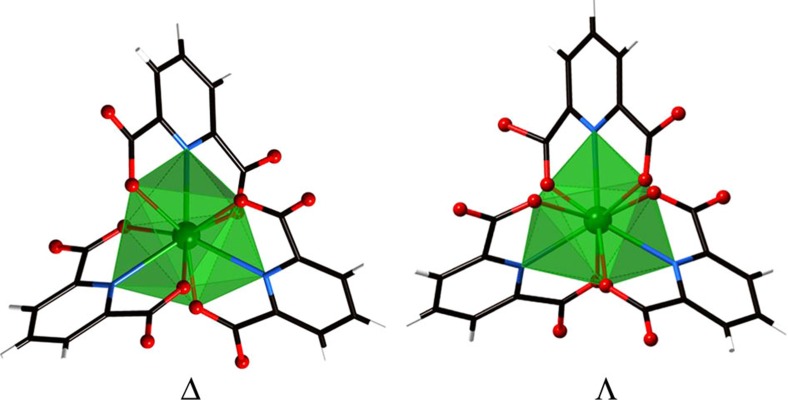
Views of the Δ and Λ enantiomers of Cf(HDPA)_3_·H_2_O. Views of the Δ and Λ enantiomers of Cf(HDPA)_3_·H_2_O showing the nine-coordinate, tricapped trigonal prismatic coordination environments of the Cf(III) ions created via chelation by three, monoprotonated, 2,6-dipicolinate ligands, HDPA^−^. The coordination environment of the Cf(III) centre in the Δ enantiomer is more distorted than in Λ enantiomer because of differences in hydrogen bonding with co-crystallized water molecules.

The reactions of hydrous ^243^AmCl_3_, ^248^CmCl_3_ and ^249^CfCl_3_ with excess 2,6-pyridinedicarboxylic acid (dipicolinic acid, DPA) at 150 °C in a 1:1 ethanol/H_2_O mixture results in the formation of crystals of An(HDPA)_3_·H_2_O (An=Am, Cm, Cf). These compounds are isomorphous and consist of nine-coordinate, tricapped trigonal prismatic An(III) ions bound by three, tridentate, monoprotonated DPA ligands. These tris-chelate complexes are necessarily chiral, and the structure, as determined by single crystal X-ray diffraction, reveals a racemic mixture of the Δ and Λ enantiomers, as expected. A view of both enantiomers is shown in Fig. 1. Crystallographic details are provided in Supplementary Table 1. Crystallographic information files (CIFs) are available in Supplementary Data 1–3.

Examination of the volume changes of the unit cells across the actinide series show the expected reduction ascribed to the actinide contraction (Supplementary Table 1). In fact, we collected high-angle diffraction data to reduce the s.d. values in the bond distances as much as possible. Inspection of the average Am–O, Cm–O, and Cf–O bond distances, as well as the An–N distances, reveals a decrease of ∼0.05 Å from Am(III) to Cf(III) as provided in Tables 1 and 2 (see also Supplementary Table 2). The expected contraction between neighbouring *f* elements is on the order of 0.01 Å. Therefore, a decrease in average bond lengths of ∼0.03 Å between Am(III) and Cf(III) is expected if a fully ionic model is imposed. The Cf–O and Cf–N bonds are slightly shorter than expected, and this might be an indicator of increased effects of covalency across the series^19^,^20^. However, this system presents a unique opportunity in that both enantiomers are present in the asymmetric unit. A comparison of the bond distance variations between enantiomers of the same element reveals that the average differences between bond lengths between the Δ and Λ enantiomers of the Cf(III) complex are on the same order as the differences observed in the bond lengths between the Am(III) and Cf(III) molecules. However, the entire structure is a part of a hydrogen bonding network, and the hydrogen bond contacts for the Δ and Λ enantiomers are not the same. Hence, these interactions cause minor distortions of the different enantiomers. If one instead compares the same enantiomers between the Am(III), Cm(III) and Cf(III) complexes, the Cf–N and Cf–O bond distances are slightly shorter than anticipated; albeit the statistical significance of these differences depends on how one treats the errors in the bond distances. It must also be kept in mind that berkelium lies between curium and californium, but preparation of the Bk(III) complex is not currently possible owing to the half-life of ^249^Bk being only 320 days.

**Figure 2:**
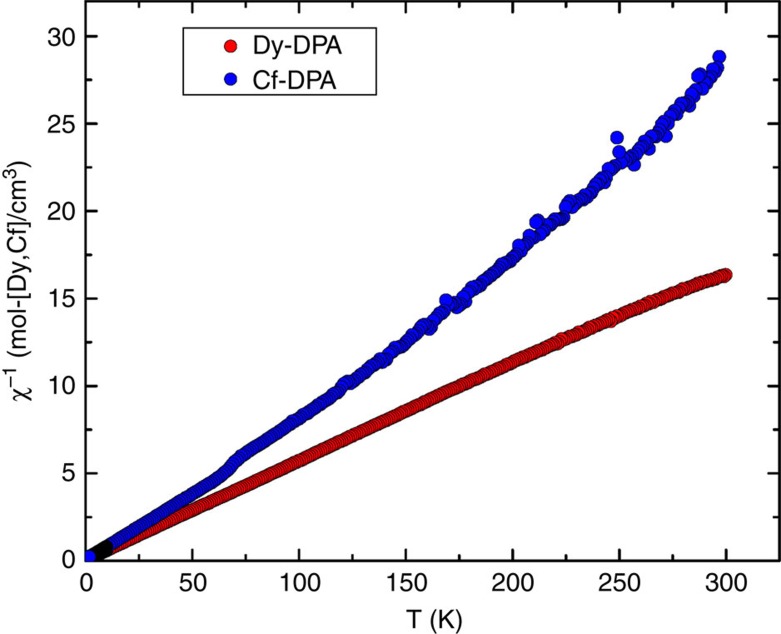
Inverse magnetic susceptibility as a function of temperature. Inverse magnetic susceptibility of polycrystalline samples of Dy(HDPA)_3_·H_2_O and Cf(HDPA)_3_·H_2_O as a function of temperature. The measured *μ*_eff_ of the Dy(HDPA)_3_·H_2_O sample reaches the theoretical free-ion moment of 10.65 *μ*_B_ expected for an *f*^9^ system, whereas the Cf(III) compound shows a reduced *μ*_eff_ of 9.3(1) *μ*_B_.

There are more convincing indicators that the electronic structure of the Cf(III) complex deviates significantly from expectations. For example, magnetic susceptibility data were collected from 300 to 1.8 K for Cf(HDPA)_3_·H_2_O under an applied field of 0.1T as shown in Fig. 2. The large temperature-independent paramagnetic effects that are indicative of low-lying excited states observed for Cf[B_6_O_8_(OH)_5_] are not found in Cf(HDPA)_3_·H_2_O. Instead the data are essentially Curie-Weiss like with a measured *μ*_eff_ of 9.3(1) *μ*_B_. This value is significantly lower than that calculated for the free-ion of 10.65 *μ*_B_ (ref. 21). Examination of previously reported magnetic moments for Cf(III) compounds shows that values of ∼9.3 *μ*_B_ are, in fact, the most commonly reported. However, most investigators have attributed these deviations from the calculated free-ion moment to experimental artefacts that result from the very small sample sizes employed in the measurements (∼1 μg of ^249^Cf), and hence large errors in the masses of ^249^Cf (refs 10, 11). It should be pointed out, however, that the quantity of ^249^Cf used in these prior studies was not determined by weighing, but rather by far more accurate radiation counting methods, and later studies conducted with tens of micrograms of ^249^Cf yielded similar values as those with much smaller quantities^10^,^11^.

**Figure 3:**
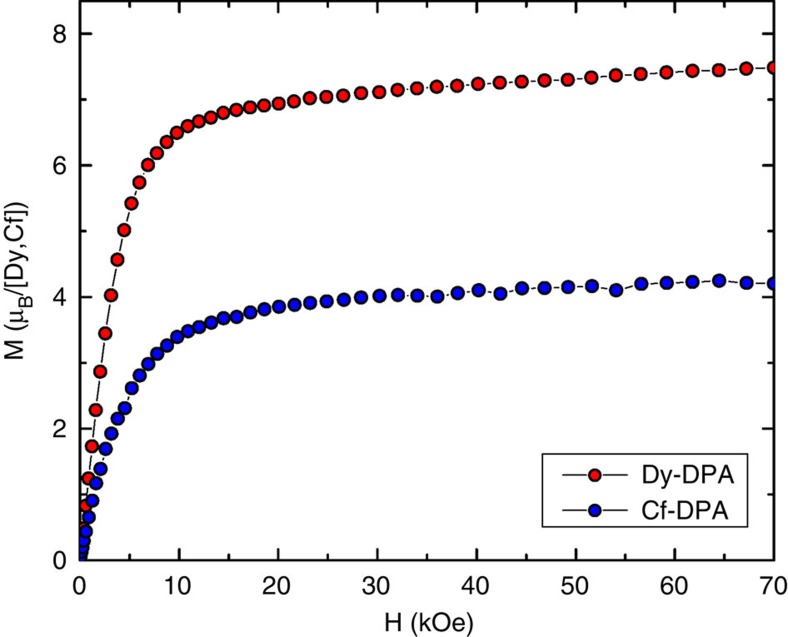
Magnetization as a function of magnetic field. Magnetization of polycrystalline samples of Dy(HDPA)_3_·H_2_O (4*f*^9^) and Cf(HDPA)_3_·H_2_O (5*f*^9^) as a function of magnetic field. The Cf(III) sample has a *μ*_sat_ value approximately half of that of the Dy(III) sample. The Dy(III) sample displays typical hard magnetism of lanthanides, whereas the Cf(III) complex shows much softer behaviour.

To demonstrate that the reduced moment measured for Cf(HDPA)_3_·H_2_O is not an artefact of the small sample size, our measurements were performed on the largest quantity of ^249^Cf ever used in a magnetic susceptibility study (2.2 mg of ^249^Cf), and the data were collected using a VSM-SQUID, which provides significantly more sensitivity than a traditional SQUID. We conclude that the earlier reports are probably correct, and that Cf(III) commonly displays reduced magnetic moments unlike isoelectronic Dy(III), whose measured *μ*_eff_ from a variety of compounds are typically close to the calculated free-ion moment^21^. In fact, we prepared Dy(HDPA)_3_·H_2_O, and measured its magnetic susceptibility in the same way, to find its exhibited moment reaches the calculated free-ion moment value, as expected. The inverse magnetic susceptibility data are compared in Fig. 2, and a large deviation between the two metals ions is clearly apparent. In addition, much like what is found in Cf[B_6_O_8_(OH)_5_], Cf(HDPA)_3_·H_2_O behaves as a much softer magnetic system than expected for an *f*-element, and magnetization versus field measurements reveals saturation of the Cf sample at approximately half of the value of the Dy complex as provided in Fig. 3.

**Figure 4:**
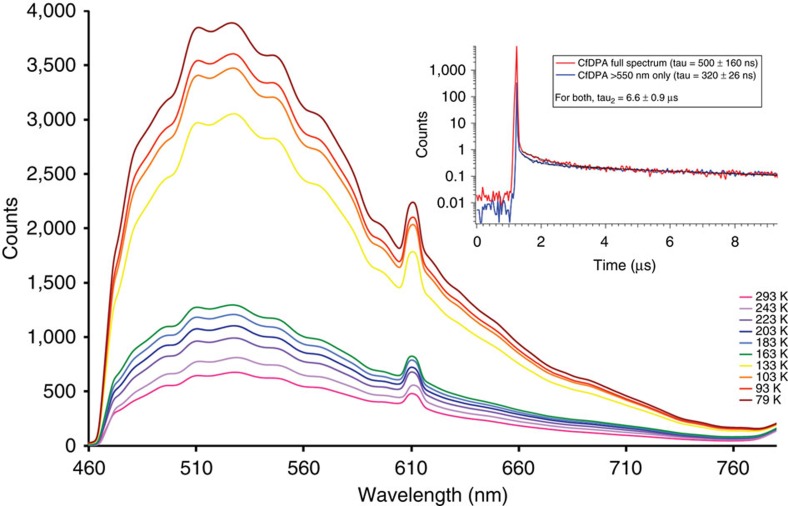
Photoluminescence spectra of Cf(HDPA)_3_·H_2_O. Photoluminescence spectra from a single crystal of Cf(HDPA)_3_·H_2_O on excitation with 420 nm light as a function of temperature. The emission from Cf(III) is centred at 525 nm, whereas the emission from the ^245^Cm(III) daughter occurs at 611 nm. Strong vibronic coupling is found for Cf(HDPA)_3_·H_2_O, but not with Cm(HDPA)_3_·H_2_O. Inset shows the decay lifetimes of 500±160 ns for Cf(III) and 320±26 ns for the Cm(III) daughter.

A deeper understanding of the electronic structure of the Cf(III) complex was achieved by measuring electronic absorption and photoluminescence spectra from single crystals. The former data are provided in Supplementary Fig. 3, and the latter in Fig. 4. We were fortunate that fairly large crystals of Cf(HDPA)_3_·H_2_O can be prepared, whereas Cf[B_6_O_8_(OH)_5_] is microcrystalline. A comparison of these spectra with An^3+^ ions in crystals with weak ligand interactions reveals that the 5*f*-5*f* transitions are abnormally broad even at 79 K. In addition, there is a very broad absorption band in the short wavelength region that cannot be attributed to 5*f*-5*f* transitions. Again, we examined earlier published data on the absorption spectra of Cf(III) compounds, and these features are typically at least an order of magnitude broader than expected^22^. Photoluminescence data, which were collected at variable-temperature using either 365 or 420 nm excitation wavelengths, show green photoluminescence centred at ∼525 nm. Historically, this photoluminescence has been assigned to the *J*=5/2 excited state transition to the *J*=15/2 ground state^8^,^23^,^24^. However, as we will delineate below, this assignment is most likely incorrect, and the actual origin is far more intriguing. Much like in Cf[B_6_O_8_(OH)_5_], strong vibronic coupling is observed, and the photoluminescence peak width at half-height is massive at ∼126 nm. In Cf(HDPA)_3_·H_2_O, the vibrational progression is more clearly resolved than in Cf[B_6_O_8_(OH)_5_], perhaps because the former is a single crystal sample.

**Figure 5:**
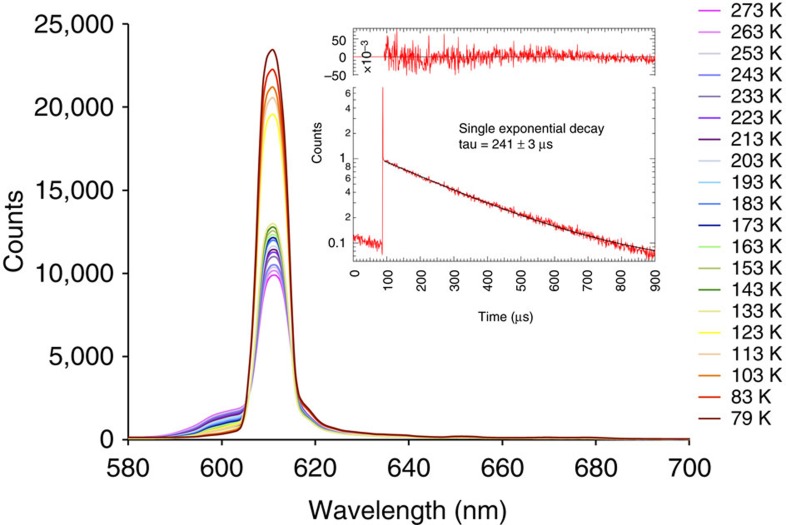
Photoluminescence spectra of ^248^Cm(HDPA)_3_·H_2_O. Photoluminescence spectra from a single crystal of ^248^Cm(HDPA)_3_·H_2_O on excitation with 420 nm light as a function of temperature. The photoluminescence from the Cm(III) complex is centred at 611 nm as found when it is doped into the Cf(III) compound in the form of the ^245^Cm daughter. The inset shows the decay lifetime of 241±160 μs, which is much longer than that found in the Cf(III) sample. This lifetime is typical of Cm(III) compounds, and is substantially shortened by the rapid creation of colour centres in the Cf(III) sample because of radiation damage.

In both Cf[B_6_O_8_(OH)_5_] and Cf(HDPA)_3_·H_2_O, photoluminescence of the daughter of ^249^Cf α decay, ^245^Cm, is also observed. In the Cf[B_6_O_8_(OH)_5_] sample, the Cm(III) photoluminescence peak is nearly as broad as that of Cf(III), leaving unanswered whether the highly electron-rich coordination environment that borate provides is solely responsible for the changes in electronic structure, or whether these features only occur because the Cm(III) is effectively being doped into the Cf(III) sample, or whether it is some combination of the two. In Cf(HDPA)_3_·H_2_O, the photoluminescence of ^245^Cm(III) at 611 nm is a sharp transition as expected for a Cm(III) complex^25^,^26^,^27^. We also prepared the pure Cm(HDPA)_3_·H_2_O complex using a ^248^Cm(III) starting material, and fully characterized this sample, including its variable-temperature photoluminescence spectra as illustrated in Fig. 5. As anticipated, the Cm(HDPA)_3_·H_2_O compound produces a single sharp emission line at 611 nm and vibronic coupling is absent. This supports the postulate that Cf(III) represents a transition point in the actinide series where emergent phenomena are apparent. Buttressing of this argument is also provided by the measured *μ*_eff_ of the Cm(III) complex being 8.0(1) *μ*_B_, which agrees well the calculated moment of 7.94 *μ*_B_ (refer to Supplementary Fig. 6), and with magnetic moments measured from other Cm(III) compounds^28^. The absorption spectrum is also typical for a Cm(III) material (refer to Supplementary Fig. 4).

### Crystal-field analysis

**Figure 6:**
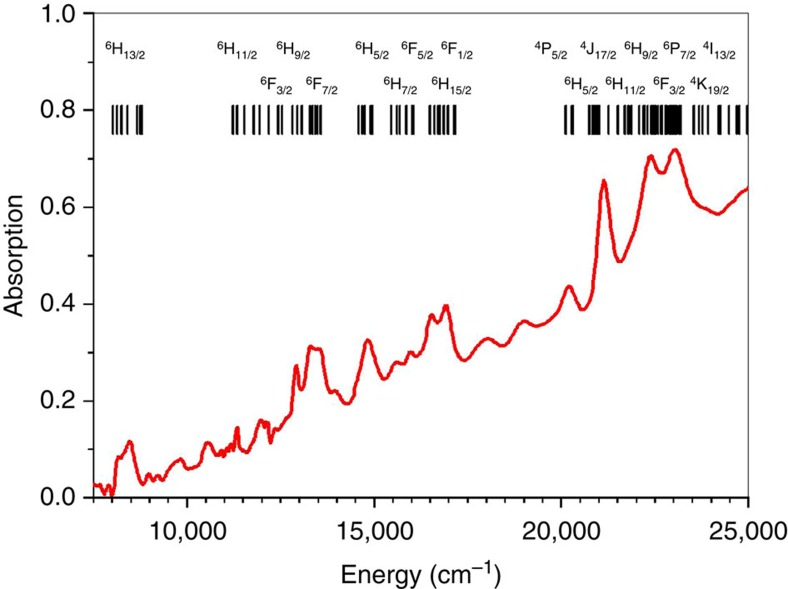
Absorption spectrum. Absorption spectrum of a single crystal of Cf(HDPA)_3_·H_2_O at 83 K. The narrower bands are due to 5*f*^9^–5*f*^9^ transitions. Calculated using an effective-operator Hamiltonian, the crystal-field splittings of the 5*f*^9^ states between 8,000 and 25,000 cm^−1^ are marked by the vertical bars along with the leading SLJ multiplets. Several weaker bands with energy levels at 9,700, 10,570, 17,900 and 18,960 cm^−1^ are not predicted from the crystal-field calculation, and are thus attributed to vibronic structures coupled to charge-transfer transition.

Crystal-field analysis allows for the electronic energy levels of Cf(III) in Cf(HDPA)_3_ to be calculated, and the resultant states are compared with the absorption spectrum as shown in Fig. 6 (ref. 29). The free-ion parameters were taken from previously obtained values for An(III) doped into LaCl_3_ crystals. Assuming *C*_3*v*_ site symmetry for Cf(III), the crystal-field parameters were first calculated using a superposition model from ligand-field theory^30^. The values of the calculated crystal-field parameters were varied proportionally from the calculated ones together with the spin-orbit coupling constant *ζ*_*5f*_ to obtain the best fit with the experimental data. Detailed analysis of the electronic energy levels and crystal-field calculations for Cf(III) and Am(III) in An(HDPA)_3_ will be reported separately. The calculated energy levels are indicated with the leading free-ion states by the vertical lines on the top of the spectrum shown in Fig. 6. These calculated levels match the experimental data quite well. The resultant crystal-field parameters are reduced by 20% from the calculated values. They are 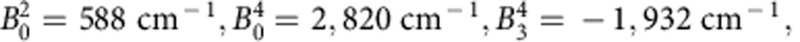 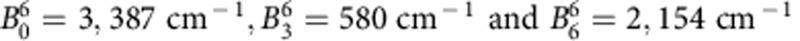. The spin-orbit coupling constant is *ζ*_*5f*_=3536, cm^−1^, which is only 1% less than that for Cf(III):LaCl_3_. The ligand-field strength (that is, *N′*_*v*_)^31^ in Cf(HDPA)_3_·H_2_O calculated from the crystal-field parameters is 1,632 cm^−1^, which is much larger than the strength of 610 cm^−1^ found for Cf(III) in a chloride environment (that is, doped into a LaCl_3_ lattice)^31^. The crystal-field splitting of the ground multiplet ^6^H_15/2_ is 824 cm^−1^. For comparison, the total splitting of ^6^H_15/2_ for the 4*f*^9^ ion Dy(III) in LaCl_3_ is only 141 cm^−1^ (ref. 32) The increased ground-state splitting would significantly reduce the magnetic susceptibility (*μ*_eff_).

A compilation of all of the electronic spectroscopy and magnetism data gathered for this work, as well as that measured from Cf[B_6_O_8_(OH)_5_]^8^, allows us to reach the following conclusions. (1) Under 365 nm excitation, the origin, or zero-phonon line of the photoluminescence of Cf(HDPA)_3_·H_2_O is above 25,000 cm^−1^ (400 nm). However, as shown in Fig. 6, this is far above the expected energy level of the *J*=5/2 emitting state (dominated by the ^4^P_5/2_ level with an energy near 20,000 cm^−1^)^31^. A large hypsochromic shift of the 5*f*-5*f* transitions is not observed in the absorption spectrum. (2) There is a substantial Stokes shift and photoluminescence band broadening with strong vibronic features. In both Cf[B_6_O_8_(OH)_5_] and Cf(HDPA)_3_·H_2_O, the californium photoluminescence band stretches more than 10,000 cm^−1^ across the narrow emission from the Cm(III) daughter centred at 611 nm. If the photoluminescence is due to a 5*f*-5*f* transition, it should have the same bandwidth as that of the 5*f*-5*f* absorption bands. In addition, no photoluminescence is observed from the corresponding Am(III) complex. This indicates that radiative 5*f*-5*f* relaxation is quenched even from the metastable 5*f*^6^ state (^5^D_1_) of Am(III). This state of Am(III) has an energy gap between ^5^D_1_ and ^7^F_6_ that is much larger than the difference between the Cf(III) ^4^P_5/2_ state and the next low-lying state ^6^F_1/2_. According to the energy-gap law^33^, if photoluminescence occurs from the ^4^P_5/2_ state of Cf(III), one should also see red ^5^D_1_ emission from the Am(III) complex.

The crystal-field strength is often used as an index of covalency in actinide compounds. Earlier work conducted by Edelstein and co-workers demonstrated that *N′*_*v*_ strongly depends on the oxidation state of the actinide ion and the valence orbitals of its ligands^34^,^35^,^36^. Summarized in Supplementary Fig. 9, the value of *N′*_*v*_ varies from 600 cm^−1^ for An(III) in chlorides and bromides to above 7,000 cm^−1^ for An(V) in fluorides. The value of *N′*_*v*_ is insensitive to the electronic configuration of ions in the same oxidation states as was realized in the An(III):LaCl_3_ and An(IV):CeF_4_ series^37^,^38^, with values of *N′*_*v*_ close to 600 and 2,000 cm^−1^, respectively, for actinides across the series. Systematically, once *N′*_*v*_ is above 2,000 cm^−1^, covalency is thought to be dominant in ion-ligand bonding. For instance, the value of *N′*_*v*_ for U(V) in (NEt_4_)UCl_6_ and (NEt_4_)UBr_6_ is between 3,000 and 4,000 cm^−1^, and these uranium complexes are considered to have predominantly covalent bonding^39^. For Cf(HDPA)_3_·H_2_O, *N′*_*v*_ is 1,623 cm^−1^, which is much larger than that for trivalent actinides in many compounds, and suggests the presence of some covalency in the bonding. This result supports the ligand hyperpolarizability arguments that we have previously put forth as well as the change in periodicity at californium^3^. The analysis of the crystal-field strength and 5*f*^9^ energy levels supports our assignment of the broadband photoluminescence to a charge-transfer transition instead of an intra 5*f* transition.

Given the above observations and analysis, there is no basis to assign the photoluminescence to a Cf(III) 5*f*-5*f* transition. This calls into question whether the assignment of the green photoluminescence has ever been correct, and we are unable to find a photoluminescence spectrum in the literature that is clearly indicative of a 5*f*-5*f* transition. The assignment most likely stems from an extension of the self-luminescence of short-lived ^242^Cm and ^244^Cm compounds, whose orange luminescence is exactly the same as that found from exciting long-lived ^248^Cm(III) compounds, where the photoluminescence is clearly 5*f* in origin. A mechanism that provides a satisfying interpretation of the spectroscopic data and is also consistent with the magnetic and thermodynamic studies is that the photoluminescence is from a ligand-to-metal charge-transfer transition that is best described as a Cf(III) to Cf(II)+h^+^ (h^+^=hole in the valence band) occurring in the energy region at ∼25,000 cm^−1^ (400 nm). Transitions that correspond to this charge-transfer are clearly apparent in the absorption spectrum (see Supplementary Fig. 1). A photon is emitted when the hole recombines with Cf(II) returning it to the Cf(III) ground state. Charge-transfer transitions are often strongly coupled to vibronic interactions. These vibronic progressions lead to significantly broader photoluminescence bands than observed for 5*f*-5*f* transitions^40^. As previously discussed, starting at californium, the divalent state becomes metastable. The mechanism of charge-transfer photoluminescence is the recombination of Cf(II) with the hole created in the ligand valence band. This occurs partially through a radiative process to the ground state of Cf(III) without complete non-radiative phonon cross relaxation into the low-lying Cf(III) excited states. To further support this mechanism of photoluminescence, we also prepared the californium sulfate fluoride, KCf_2_(SO_4_)_2_F_3_, where it would be expected that creating a hole in the ligands would be energetically challenging. This compound is not luminescent, even at low temperatures.

## Discussion

All of the data disclosed in this report support Cf(III) complexes displaying emergent phenomena that cannot be predicted from either extrapolating from its isoelectronic lanthanide analogue, Dy(III), or from other trivalent actinides^3^,^8^,^41^. A juxtaposition of factors that include the relative ease of reducing Cf(III) to Cf(II) and presence of available 5*f*, 6*d*, 7*s* and 7*p* acceptor orbitals creates an unusually large ligand-field strength that significantly alters the electronic properties anticipated for an *f*-element^8^. The atypical features include a reduced magnetic moment and vibronically coupled, charge-transfer-based photoluminescence. Reduction in the magnetic moment of transition metal and *f*-element complexes can occur via a variety of mechanisms that include the size of the crystal-field splitting of the ground state, spin-orbit coupling, and delocalization of electrons from the metal ions onto the ligands (that is, covalent bonding and ligand-field effects)^42^,^43^. Crystal-field theory is based purely on electrostatics, but is adequate for lanthanides because the 4*f* orbitals lie within the xenon core and are effectively nonbonding. We have shown here that the moment reductions can arise from several factors. In the case of the Cm(III) complex, the magnetic susceptibility is normal for a 5*f*^7^ system^28^. However, for the Cf(III) complex the reduced magnetic moment is caused by ligand-field effects on the 5*f* electrons that are quite pronounced as indicated by a large ligand-field strength and notable splitting of the ground state.

We offer the following hypothesis that both explains these observations and predicts the outcome of future studies with late actinides: the alterations in californium's physical and chemical properties are caused by ligand-field effects that manifest because of the relative ease with which formally Cf(III) can be reduced to Cf(II)^13^. Fajan's Rules also support that the smaller size of the Cf(III) ion relative to earlier actinides should create greater polarization of the electron density from the ligands to the metal centre^44^,^45^. Therefore, these perturbations will become more pronounced with later actinides because the 2+ oxidation state becomes progressively more stable and the metal ions continue to diminish in size. This combination of thermodynamics and ion size overrides the minor contraction of the 5*f* orbitals in the actinide series. In fact, even in biphasic extraction studies used for separating 5*f* elements from one another, a marked break is observed between Cm(III) and Cf(III) that supports californium's chemistry deviating from earlier actinides regardless of whether it is in the solid-state or a coordination complex in solution^46^. While the data are still scant, californium consistently shows larger binding constants than would be anticipated^47^. Gathering the data needed to bolster this hypothesis will require renewed production of einsteinium and heavier elements. We predict that complexes of einsteinium, fermium and mendelevium will show greater perturbations from their isoelectronic lanthanide analogues than even californium because of the increasing stability of the divalent state and the involvement of valence orbitals in bonding.

## Methods

### Experimental

^243^Am (*t*_1/2_ =7.38 × 10^3^ years) and ^248^Cm (*t*_1/2_ =3.48 × 10^5^ years) represent potential health risks owing to their *α* and *γ* emission, and the emission of their daughters. ^243^Am decays to ^239^Np (*t*_1/2_=2.35 days) which is a β- and γ-emitter; ^248^Cm decays to ^244^Pu (*t*_1/2_=8.08 × 10^7^ years) as well as undergoing spontaneous fission (which accounts for 8.3% of its decay) releasing a large flux of neutrons that can have a specific activity of ∼100 mRem h^−1^ for the sample size used. ^249^Cf (*t*_1/2_=351 years; specific activity=4.1 Ci/g) represent a serious external hazard because of its *γ* (0.388 MeV) emission. ^249^Cf decays to ^245^Cm (*t*_1/2_=8,500 years), which also has a high specific activity. All studies with transuranium elements were conducted in a laboratory dedicated to these studies. This laboratory is equipped with HEPA-filtered hoods and negative pressure glove boxes that are ported directly into the hoods. A series of counters continually monitor radiation levels in the laboratory. The laboratory is licensed by the State of Florida (an NRC-compliant state). All experiments were carried out with approved safety operating procedures. All free-flowing solids are worked with in glove boxes, and products are only examined when coated with either water or Krytox oil and water. The ^249^Cf sample used produces 1.7 R h^−1^ at 40 mm, and ∼10 R h^−1^ at contact, and therefore represents a serious external hazard that required the experiments to be carefully choreographed to minimize exposure times. Thick lead sheets and long lead vests were used as much as possible to shield researchers from the *γ-* emission.

### Syntheses

All Ln(HDPA)_3_·H_2_O and An(HDPA)_3_·H_2_O compounds were prepared using 5 mg of the appropriate *f* element, which was then combined with a fivefold excess of DPA in 100 μl of a 1:1 mixture of ethanol and water. The resultant reaction mixture was heated in a PTFE-lined Parr 4749 autoclave with a 10 ml internal volume for 4 h at 150 °C, and then slowly cooled to 23 °C over a 12-h period. While reactions with the lanthanides were conducted in a standard muffle furnace in a hood, the furnaces for heating the ^243^Am and ^249^Cf autoclaves were inside a negative-pressure glovebox and were surrounded by thick lead sheets. The reactions result in the formation of crystals of the appropriate colours for the *f* elements with block and columnar habits (see Supplementary Figs 2,5 and 8).

### Crystallographic studies

Single crystals of the Ln_2_(HDPA)_6_·2H_2_O (see Supplementary Table 3) and An_2_(HDPA)_6_·2H_2_O compounds were glued to Mitogen mounts with epoxy and optically aligned on a Bruker D8 Quest X-ray diffractometer using a digital camera. Initial intensity measurements were performed using a IμS X-ray source (MoKα, *λ*=0.71073 Å) with high-brilliance and high-performance focusing multilayered optics. Standard software was used for determination of the unit cells and data collection control. The intensities of reflections of a sphere were collected by a combination of multiple sets of exposures (frames). Each set had a different *ϕ* angle for the crystal and each exposure covered a range of 0.5° in *ω*. A variety of data collection strategies were employed including standard hemispheres, and more complex data sets with higher angles and greater degrees of redundancy. The SAINT software was used for data integration including Lorentz and polarization corrections. The structure was solved by direct methods and refined on F^2^ by full-matrix least squares techniques using the program suite SHELX (Supplementary Figs 1 and 7). Parameters for Am, Cm and Cf are not present in the SHELX software and have to be input manually. Solutions were checked for missed symmetry using PLATON^48^.

### UV–vis-NIR and photoluminescence spectroscopy

UV–vis-NIR and photoluminescence data were acquired from single crystals using a Craic Technologies microspectrophotometer. Crystals were placed on quartz slides under Krytox oil, and the data were collected from 200 to 1,700 nm. The exposure time was auto optimized by the Craic software. Photoluminescence data were acquired using the same microspectrophotometer with an excitation wavelength of 365 or 420 nm (Figs 4 and 5; see also Supplementary Figs 2,3 and 5). Temperature control was achieved by using a Linkam temperature control stage. Raman measurements were also attempted, but these were impeded by the self-luminescence and rapid decomposition of the sample in laser beam.

### Life-time measurements

Time-correlated single-photon counting (TCSPC) photoluminescence measurements were carried out using a femtosecond laser system. A regeneratively amplified titanium:sapphire laser system (Spectra Physics Tsunami coupled to Spitfire amplifier) produced laser pulses centred at 800 nm with a duration of 100 fs. The fundamental output was frequency doubled and subsequently attenuated to produce a 10 μJ per pulse, 400 nm excitation source for the TCSPC experiment. The photoluminescence was isolated from residual excitation pulses using long-pass filters, and detected using an avalanche photodiode (Quantique) coupled to a single photon-counting unit (Becker Hickl). The low-energy portion of the photoluminescence was isolated using a 550-nm long-pass filter. The resulting lifetime data were fit to a biexponential decay function (Figs 4 and 5).

### Magnetic measurements

Magnetic measurements were performed on polycrystalline samples that were encapsulated in tightly closed PTFE sample holders with a Quantum Design SQUID magnetometer MPMS-XL or a VSM-SQUID MPMS. DC magnetic susceptibility measurements were carried out in an applied field of 0.100 T in the 1.8–300 K temperature range. Field-dependent magnetization was recorded at 1.8 K under an applied magnetic field that was varied from 0 to 7T. The data were corrected for the diamagnetic contribution from the sample holder and constituent elements (Figs 2 and 3).

## Author contributions

S.K.C., J.N.C., J.T.S., M.J.P. and T.E.A.-S. conceived, designed and carried out the synthetic and crystallographic experiments. M.V., D.A.D., M.A.S. and A.E.D. aided in the development of the bonding concepts. S.K.C., J.T.S. and J.H.H. carried out variable-temperature absorption and photoluminescence experiments. G.L. analysed all electronic spectroscopy experiments. S.K.C., T.E.A.-S., N.K., K.D., A.G. and R.E.B. designed and carried out the magnetic susceptibility experiments. T.B.G. and K.L.K. carried out the photoluminescence life-time measurements. A.A.A. carried out PXRD measurements. S.M.V.C. prepared and manipulated the original stock of ^249^Cf at ORNL. All authors discussed and co-wrote the manuscript.

## Additional information

**Accession codes**: The X-ray crystallographic coordinates for structures reported in this Article have been deposited at the Cambridge Crystallographic Data Centre (CCDC), under deposition numbers CCDC 1028642, 1028643 and 1028646. These data can be obtained free of charge from The Cambridge Crystallographic Data Centre via www.ccdc.cam.ac.uk/data_request/cif.

**How to cite this article:** Cary, S. K. *et al.* Emergence of californium as the second transitional element in the actinide series. *Nat. Commun.* 6:6827 doi: 10.1038/ncomms7827 (2015).

## Supplementary Material

Supplementary Figures, Supplementary Tables and Supplementary References Supplementary Figures 1-9, Supplementary Tables 1-3 and Supplementary References

Supplementary Data 1 CIF file for Am complex

Supplementary Data 2 CIF file for Cm complex

Supplementary Data 3 CIF file for Cf complex

## Figures and Tables

**Table 1:** Selected Bond lengths (Å) for An(HDPA)_3_ (An=Am, Cm, Cf) complexes

	**Am Δ**	**Cm Δ**	**Cf Δ**		**Am Λ**	**Cm Λ**	**Cf Λ**
O13	2.472 (3)	2.462 (3)	2.455 (4)	O1	2.507 (3)	2.496 (3)	2.476 (4)
O14	2.491 (3)	2.483 (3)	2.443 (4)	O2	2.441 (3)	2.433 (3)	2.413 (4)
O15	2.430 (3)	2.417 (3)	2.387 (4)	O3	2.405 (3)	2.389 (3)	2.363 (3)
O16	2.468 (3)	2.459 (3)	2.422 (3)	O4	2.512 (3)	2.501 (3)	2.476 (3)
O17	2.516 (3)	2.508 (3)	2.494 (4)	O5	2.494 (3)	2.480 (3)	2.441 (3)
O18	2.499 (3)	2.481 (3)	2.411 (4)	O6	2.457 (3)	2.448 (3)	2.417 (4)
N1	2.550 (4)	2.533 (4)	2.512 (4)	N2	2.556 (3)	2.520 (4)	2.518 (4)
N3	2.551 (4)	2.536 (4)	2.508 (4)	N4	2.531 (4)	2.535 (4)	2.506 (4)
N5	2.591 (3)	2.581 (4)	2.545 (4)	N6	2.573 (4)	2.569 (4)	2.526 (4)

**Table 2:** Comparison of bond lengths between the Δ and Λ enantiomers of An(HDPA)_3_.

	**Am Δ (Å)**	**Cm Δ (Å)**	**Cf Δ (Å)**	**Am Λ (Å)**	**Cm Λ (Å)**	**Cf Λ (Å)**
Longest An-O	2.515 (4)	2.509 (4)	2.494 (4)	2.519 (4)	2.501 (4)	2.477 (4)
Shortest An-O	2.431 (4)	2.416 (4)	2.386 (4)	2.401 (4)	2.388 (4)	2.363 (4)
Average An-O	2.482 (4)	2.468 (4)	2.436 (4)	2.468 (4)	2.457 (4)	2.431 (4)
Longest An-N	2.589 (4)	2.582 (4)	2.545 (4)	2.573 (4)	2.566 (4)	2.526 (4)
Shortest An-N	2.551 (4)	2.532 (4)	2.508 (4)	2.532 (4)	2.519 (4)	2.506 (4)
Average An-N	2.564 (4)	2.550 (4)	2.522 (4)	2.554 (4)	2.540 (4)	2.517 (4)
