# Supplementary material for: Emergence of californium as the second transitional element in the actinide series
Source: Nat Commun. 2015 Apr 16;6:6827. doi: 10.1038/ncomms7827 (PMC4410632; doi:10.1038/ncomms7827)
Supplement: Supplementary Figures, Supplementary Tables and Supplementary References — Supplementary Figures 1-9, Supplementary Tables 1-3 and Supplementary References [file ncomms7827-s1.pdf]

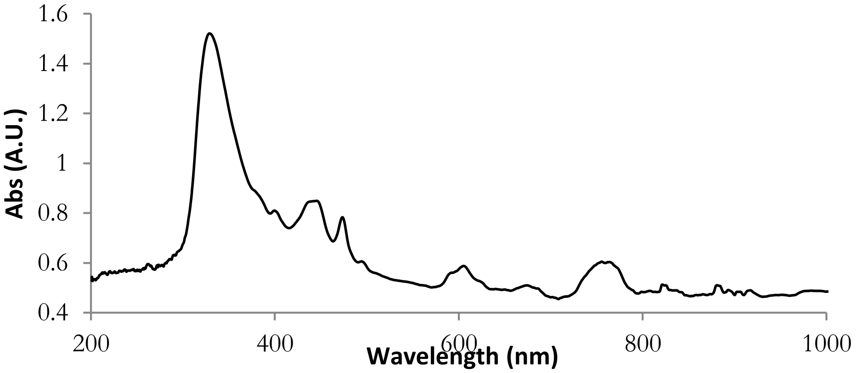

Supplementary Figure 1: Absorption spectrum taken from a single crystal of Cf(HDPA)$_3$·H_2O. The intense transition at 350 nm is a combination of the absorption of the π-π* of the DPA ligand and a LMCT band in the near UV region. The transitions at longer wavelengths are *f*-*f*, and are much broader than typically found. Below 330 nm, the spectrum is truncated by the cryostat window.

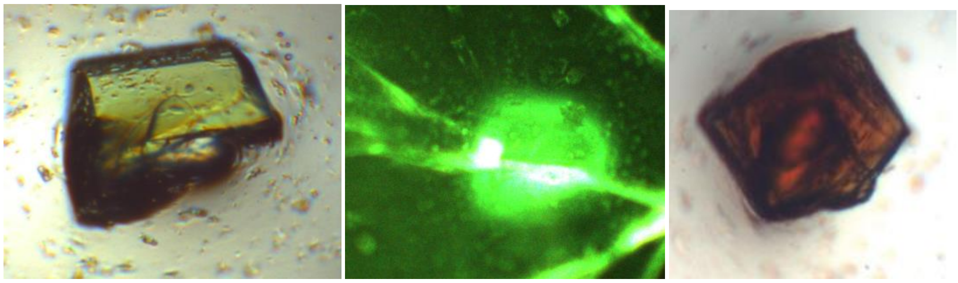

Supplementary Figure 2: Photograph of a single crystal of Cf(HDPA)$_3$·H_2O at room-temperature (left). The crystal is approximately 50 μm in size. Middle: Green photoluminescence from the crystal while frozen in a glass at 79 K. The self-luminescence (green glow) from the sample can also be observed at room-temperature when several milligrams of material are present. Right: Formation of color centers in the crystals after one week as the result of radiation damage.

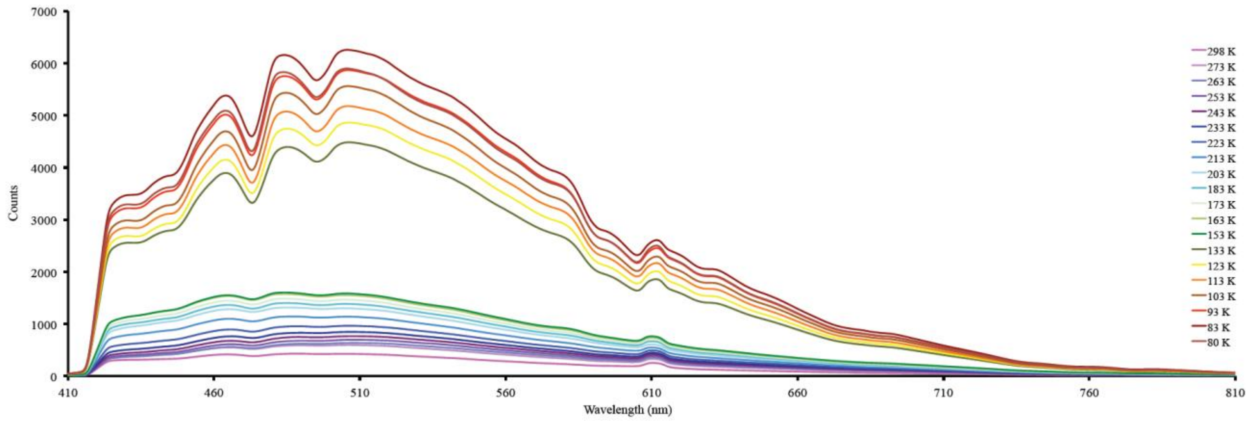

Supplementary Figure 3: Photoluminescence spectra from a single crystal of Cf(HDPA)$_3$·H_2O upon excitation with 365 nm light as a function of temperature. The photoluminescence from Cf(III) is centered at 525 nm; whereas the photoluminescence from the ^{245}Cm(III) daughter occurs at 611 nm.

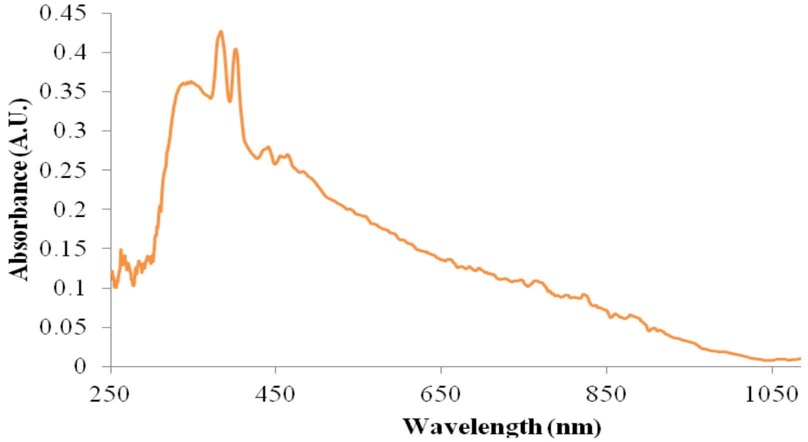

Supplementary Figure 4: Absorption spectrum taken from a single crystal of $^{248}Cm(HDPA)_3 \cdot H_2O$.

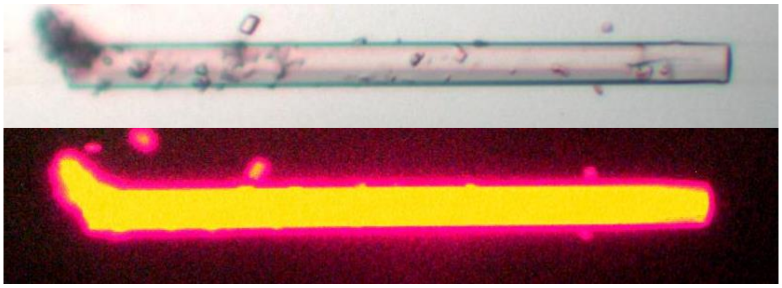

Supplementary Figure 5: (top) Photograph of a single crystal of $^{248}Cm(HDPA)_3{\cdot}H_2O$ at room-temperature. The pink coloration is false-color. The crystals are very pale yellow (essentially colorless). Bottom: Photoluminescence of the crystal upon irradiation with 420 nm light.

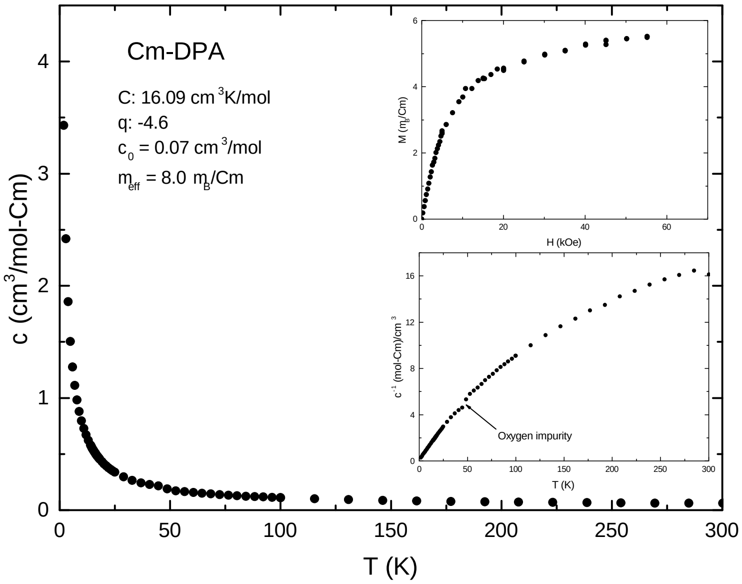

Supplementary Figure 6: Summary of the magnetic properties of $^{248}Cm(HDPA)_3 \cdot H_2O$.

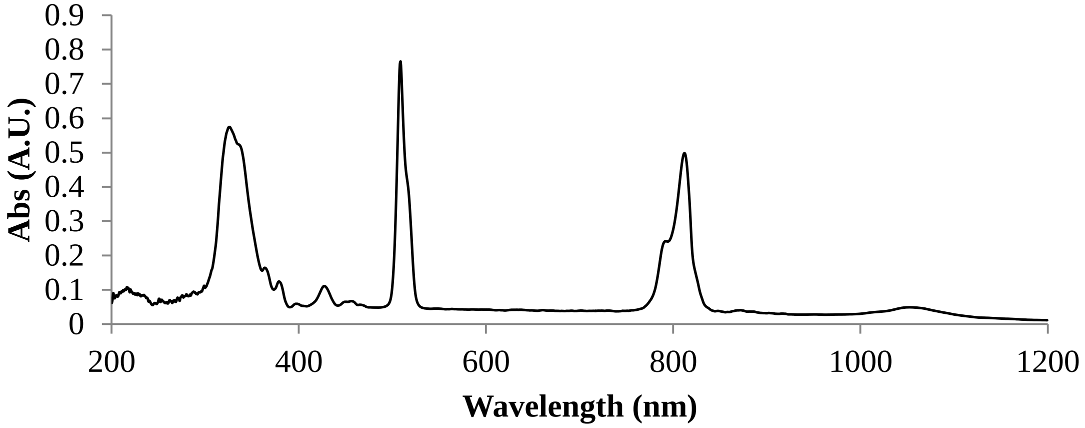

Supplementary Figure 7: Absorption spectrum taken from a single crystal of $^{243}Am(HDPA)_3{\cdot}H_2O$.

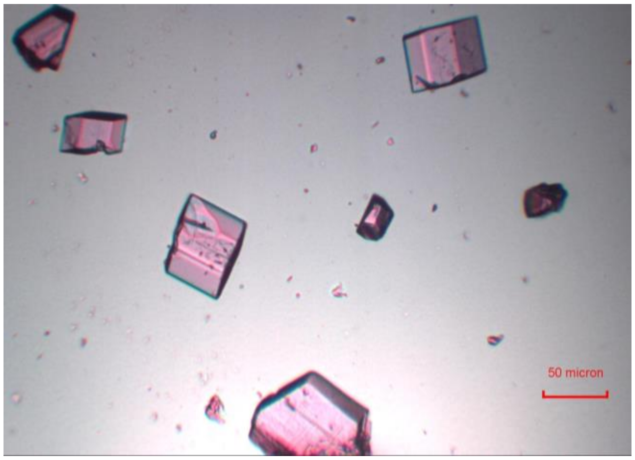

Supplementary Figure 8: Photograph of single crystals of $^{243}Am(HDPA)_3{\cdot}H_2O$. These crystals are not luminescent.

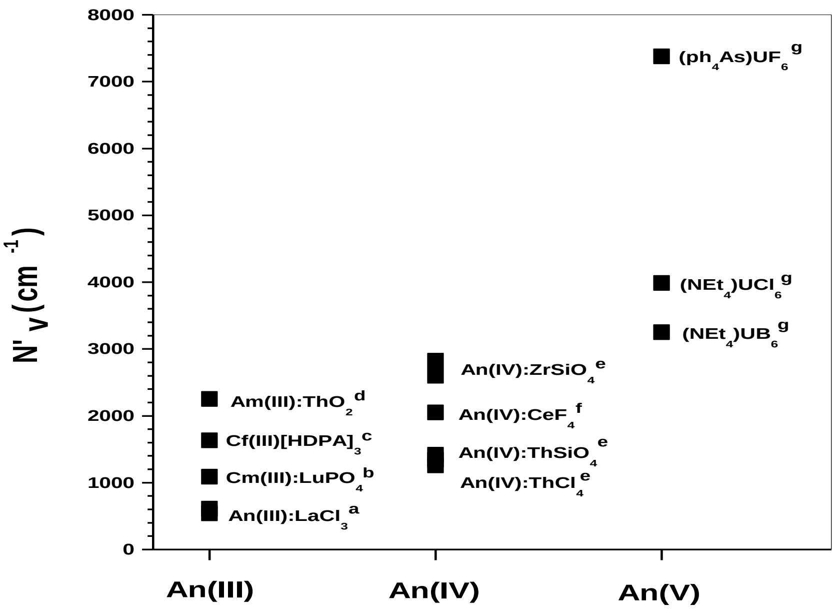

Supplementary Figure 9: Comparison of crystal-field strength N'_v for 3+, 4+, and 5+ actinide ions in various compounds. [a](Ref. 1), [b](Ref. 2), [c]This work, [d](Ref. 3), [e](Ref. 4), [f](Ref. 5), [g](Ref. 6).

Supplementary Table 1: Selected Crystallographic Information for $An(HDPA)_3 \cdot H_2O$.[a]

Compound	$Am_2(HDPA)_6 \cdot 2H_2O$	$Cm_2(HDPA)_6 \cdot 2H_2O$	$Cf_2(HDPA)_6 \cdot 2H_2O$
Formula Mass	1514.67	1524.67	1526.67
Color and habit	Pink, block	Colorless, rod	Green, block
Space group	$P\bar{1}$	$P\bar{1}$	$P\bar{1}$
a (Å)	11.8381(9)	11.8146(10)	11.7542(4)
b (Å)	13.3788(10)	13.3852(12)	13.4315(5)
c (Å)	15.1601(11)	15.1717(13)	15.1978(6)
α (°)	91.0206(12)	90.9375(17)	91.1180(12)
β (°)	95.2167(12)	95.3161(17)	95.9110(11)
γ (°)	103.1085(12)	103.1110(17)	103.1070(12)
V (Å^3)	2327.0(3)	2324.9(3)	2322.17(15)
Z	2	2	2
T (K)	100(2)	100(2)	100(2)
λ (Å)	0.71073	0.71073	0.71073
Maximum 2θ (°)	30.608	27.630	30.626
ρ_{calc} (g/cm^3)	2.162	2.178	2.183
μ (Mo $K\alpha$) (cm^{-1})	33.74	35.84	37.26
$R(F)$ for $F_o^2 > 2\sigma(F_o^2)^b$	0.0382	0.0353	0.0386
$R_w(F_o^2)^c$	0.0871	0.0779	0.0896

[a] The formula used through this manuscript has been divided by 2 throughout for the sake of simplicity because both enantiomers are present in the asymmetric unit.

[b] $R(F) = \sum \| F_o | - | F_c \| / \sum | F_o |$

[c] $R_w\left(F_o^2\right) = \left[\sum \left[w\left(F_o^2 - F_c^2\right)^2 \right] / \sum wF_o^4 \right]^{1/2}$

Supplementary Table 2: Average An-N and An-O bond lengths in the An(HDPA)$_3$·H_2O compounds.

Element	Nitrogen (Å)	Oxygen (Å)
^{243}Am	2.559(4)	2.475(4)
^{248}Cm	2.545(4)	2.462(4)
^{249}Cf	2.519(4)	2.433(4)

Supplementary Table 3: Crystallographic Information for $Ln(HDPA)_3 \cdot H_2O$.

Compound	$Gd_2(HDPA)_6 \cdot 2H_2O$	$Dy_2(HDPA)_6 \cdot 2H_2O$
Formula Mass	1343.17	1353.67
Color and habit	Colorless, rod	Colorless, rod
Space group	$P\bar{1}$	$P\bar{1}$
a (Å)	11.7720(11)	11.6348(5)
b (Å)	13.4678(12)	13.4967(6)
c (Å)	15.2508(14)	15.0709(7)
α (°)	90.7215(18)	90.7546(9)
β (°)	96.0056(19)	95.6855(9)
γ (°)	103.1656(19)	103.1939(9)
V (Å^3)	2339.8(4)	2291.2(1)
Z	2	2
T (K)	100(2)	100(2)
λ (Å)	0.71073	0.71073
Maximum 2θ (°)	27.542	27.427
ρ_{calc} (g/cm^3)	1.906	1.962
μ (Mo $K\alpha$) (cm^{-1})	29.11	33.40
$R(F)$ for $F_o^2 > 2\sigma(F_o^2)^b$	0.0403	0.0517
$R_w(F_o^2)^c$	0.1049	0.1259

Supplementary References

1. W. T. Carnall, A systematic analysis of the spectra of trivalent actinide chlorides in D 3h site symmetry. *J. Chem. Phys.* **96**, 8713-8726 (1992).

2. J. Sytama, K. M. Murdoch, N. M. Edelstein, L. A. Boatner and M. M. Abraham, Spectroscopic studies and crystal-field analysis of Cm^{3+} and Gd^{3+} in $LuPO_4$. *Phys. Rev. B* **52** 12668-12676 (1995).

3. S. Hubert, P. Thouvenot, and N. M. Edelstein, Spectroscopic studies and crystal-field analyses of Am^{3+} and Eu^{3+} in the cubic-symmetry site of ThO_2. *Phys. Rev. B* **48**, 5751 (1993).

4. J. C. Krupa, Spectroscopic properties of tetravalent actinide ions in solids. *Inorg. Chim. Acta* **139**, 223-241 (1987).

5. G. K. Liu, W. T. Carnall, G. Jursich, and C. W. Williams, Analysis of the crystal-field spectra of the actinide tetrafluorides. II. AmF_4, CmF_4, Cm^{4+}:CeF_4, and Bk^{4+}:CeF_4 *J. Chem. Phys.* **101**, 8277-8289 (1994).

6. N. Edelstein, D. Brown, and B. Whittaker, Covalency effects on the ligand field splittings of octahedral 5f7 compounds. *Inorg. Chem.* **13**, 563-567 (1974).
